# Innovations in Nursing Education, Practice and Research: Emphasising Health Literacy

**DOI:** 10.3390/nursrep16020075

**Published:** 2026-02-21

**Authors:** Antonio Martínez-Sabater, Elena Chover-Sierra, Carlos Saus-Ortega

**Affiliations:** 1 Nursing Care and Education Research Group (GRIECE), GIUV2019-456, Nursing Department, Universitat de Valencia, 46010 Valencia, Spain; 2 Care Research Group (INCLIVA), Hospital Clínico, Universitat de Valencia, 46010 Valencia, Spain; 3 Department of Nursing, Faculty of Health Sciences, Research Group in Care GRUPAC, University of La Rioja, 26006 Logroño, Spain; 4 Internal Medicine, Consorci Hospital General Universitari de Valencia, 46014 Valencia, Spain; 5 Nursing School La Fe, Adscript Center of Universidad de Valencia, 46026 Valencia, Spain

Health literacy (HL) is a key determinant of quality, equity, and person-centred healthcare. Defined as the ability to access, understand, evaluate, and apply health information to make appropriate decisions, HL directly influences self-care, patient safety, clinical effectiveness, and health outcomes. In nursing, this competence is especially relevant, as practice involves ongoing patient education, communication, and support across the continuum of care [1,2,3,4,5,6].

The main objective of this Special Issue is to critically analyse how HL and digital health literacy (DHL) can be systematically integrated into nursing education, clinical practice, and research in an evidence-based manner. Specifically, it aims to synthesise empirical evidence and recent systematic reviews on educational programmes to improve them among nursing students and professionals; identify pedagogical, methodological, and evaluative innovations that foster communicative, critical, and digital competencies for person-centred care; and propose conceptual frameworks and strategic directions to guide future educational, research, and organisational initiatives, ultimately promoting safer, more equitable, inclusive, and higher-quality care across healthcare contexts.

HL is strategically significant in nursing education. To communicate effectively with patients, families, and communities, future professionals must be able to interpret complex information and translate it into clear, accessible, and culturally relevant messages. However, evidence indicates that nursing curricula still lack systematic integration of HL content, limiting the preparation of future-generation nurses [4,6,7]

Empirical studies underscore the importance of addressing this gap. Randomised trials show that targeted educational programmes significantly improve nursing students’ HL compared with control groups, reinforcing the role of HL training in developing competent health educators. Recent studies have evaluated structured HL-focused educational programmes, clearly describing their content, format, and outcomes. For example, a randomised controlled trial involving 206 nursing students found that a four-week HL education programme significantly increased HL levels compared with controls, supporting its effectiveness within undergraduate curricula [8]. Similarly, combined HL and intercultural competence interventions delivered through classroom teaching and supervised clinical practice demonstrated significant improvements in students’ self-efficacy, knowledge, and beliefs related to HL [9]. Evidence from international contexts, such as structured HL training among nursing students in Tanzania, also reported significant gains in HL-related knowledge, skills, and attitudes following pre–post-interventions [10].

Overall, these programmes share explicit didactic content on accessing, understanding, and communicating health information, blended theoretical–practical formats, and cognitive and attitudinal outcomes, providing robust evidence of the effectiveness of HL education in nursing. Reviews further indicate that interventions targeting eHealth literacy enhance access to and use of digital resources, supporting more informed decision-making and appropriate use of health services [4,8,11]. HL should be regarded as a core cross-cutting competence in nursing education, clinical practice, and health policy, as nurses play a central role in adapting communication, facilitating informed decision-making, and supporting patients across diverse care contexts.

Systematic reviews indicate that health literacy (HL) remains underrepresented in nursing curricula, typically beyond general patient education strategies [7,12]. Empirical studies consistently show that many nursing students have notable knowledge gaps regarding HL, despite some ability to apply educational strategies [13,14]. When curricula include explicit HL training, significant improvements are observed even after short periods of guided instruction [8]. Longitudinal studies, including those in Spain, show that although HL levels increase throughout nursing programmes, a substantial proportion of students remain insufficiently prepared, highlighting the need for early and sustained curriculum integration [15]. Evidence-based educational strategies include integrated curricular approaches and active teaching methods. Programmes embedding HL use multimodal strategies, such as analysing patient education materials, creating simplified resources, and conducting guided communication exercises, leading to improvements in practical skills and professional awareness [7].

In eHealth literacy, interventions tailored to specific groups significantly enhance digital literacy (as measured with validated tools such as eHEALS), emphasising the value of critical evaluation and the practical application of digital resources [11]. To inform future education and research, various theoretical frameworks have been proposed. Integrated HL models outline competencies in accessing, understanding, evaluating, and applying health information, providing a basis for structuring programmes and linking HL to health outcomes and professional practice [3]. Frameworks linking HL to psychological factors, such as self-efficacy and decision-making, support training strategies that foster both knowledge and critical behavioural skills, thereby enhancing nursing practice and research [16].

In clinical practice, HL also influences the complexity of care. Patients with lower HL levels present more nursing diagnoses, interventions, and clinical risks, highlighting its direct impact on care planning and outcomes [17]. In this context, validating the nursing outcome of health literacy behaviour represents an important advance, enabling systematic assessment of individuals’ ability to navigate health information and healthcare systems [18].

DHL, critical use of social media, evaluation of online resources, and innovative educational environments are reshaping contemporary nursing practice. Integrating these elements not only modernises training but also allows nurses to address clinical and social challenges more effectively. Accordingly, this monograph provides a multidimensional and up-to-date perspective on HL as a driver of transformation in nursing education and practice, thereby promoting a more competent, equitable, and person-centred model of care that is responsive to the needs of individuals and communities [19,20,21].

In the context of the significant digitalization of health systems, it is essential to clarify the concepts of DHL and innovative educational environments. DHL can be defined as the ability to search for, access, understand, critically evaluate, and apply health-related information from digital environments, including websites, social media, mobile applications, telemedicine platforms, and other electronic resources, to make informed and confident health decisions [16,22]. This construct extends traditional HL by incorporating digital competencies, critical thinking, and skills to manage information overload and online misinformation. Evidence shows that adequate DHL levels are associated with more effective use of digital health resources, greater autonomy and empowerment of patients and professionals, improved decision-making, and more appropriate use of health services. Conversely, low DHL is linked to increased vulnerability to misinformation, poorer adherence to recommendations, and less favourable health outcomes [4,19].

Innovative educational environments refer to training contexts that integrate digital technologies and active methodologies, such as case-based learning; clinical simulations; and experiential or reflective learning, interactivity, and student-centred approaches, to foster the development of complex competencies transferable to professional practice. Evidence in nursing education indicates that these environments facilitate competencies in DHL, critical thinking, and clinical communication, particularly when they include activities focused on the critical evaluation of digital information and its application in real care settings [4,19]. Accordingly, integrating DHL within innovative educational environments represents a key strategy for preparing future nurses to address the informational, technological, and ethical challenges of contemporary healthcare.

This Special Issue aims to provide an in-depth analysis of how HL can be systematically integrated into nursing education and professional practice. In an increasingly complex healthcare context, characterised by digitalisation, misinformation, climate-related health challenges, and growing psychosocial needs, HL emerges as a strategic competence for both professionals and patients. The included studies underscore the need to renew traditional educational approaches and to conceptualise HL as a core dimension of nursing education with direct implications for communication, shared decision-making, and health system adaptability. The pedagogical innovations described positively impact critical, reflective, and communicative competencies, ranging from introductory courses to structured frameworks for care narration and validated tools to assess digital literacy in social media contexts. These findings support a more participatory and interdisciplinary educational model that prepares nurses to be confident, autonomous, and capable of delivering person-centred care.

Taken together, the studies in this Special Issue identify HL and DHL as core competencies linking educational preparedness with clinical effectiveness and equity. These include DHL Competence Labs delivered through blended learning approaches, incorporating guided searches, fact-checking, and critical evaluation of web-based and social media content, supported by validated tools such as the adapted eHEALS [19]. Introductory courses focused on professional identity can also integrate specific HL objectives, such as ethics, clinical communication, and shared decision-making, using active methodologies, case-based learning, and early clinical exposure to foster professional commitment and role understanding [20]. Additionally, mental HL and stigma reduction modules addressing early problem recognition, care pathway planning, and non-stigmatising language are particularly effective when implemented through interview simulations and role-play [23,24]. Integrating planetary health literacy through problem-based learning on climate change and health, patient and community communication, and adaptation of educational materials for diverse audiences further broadens students’ critical and social perspectives [23]. Finally, optimising learning environments using student satisfaction scales allows continuous monitoring and refinement of the contexts in which HL and DHL competencies are developed [25].

In clinical and continuing education contexts, integrating targeted educational interventions into care practice is essential for consolidating and applying HL and DHL competencies. Structured training in clinical communication, based on the RECAP framework, enhances care narration, the use of clear language, the application of the teach-back technique, and uncertainty management through deliberate practice and systematic feedback [18]. Similarly, ethics seminars and debriefing sessions addressing moral distress promote ethical resilience, clinical dilemma analysis, and reflective supervision, supporting safe and person-centred communication practices [26]. For specific populations, gerontological HL modules incorporating supervised intergenerational contact, adapted educational materials, and ageism-sensitive communication assessment have proven effective in improving care quality for older adults [27]. Likewise, systematic HL assessment during pregnancy, combined with tailored educational materials and care pathways—using clear language, multimedia resources, and easy-to-read formats—supports personalised health education and reduces inequalities in maternal and child care [28]. Finally, in perioperative settings, self-management education programmes integrating the teach-back technique and supervised routines improve clinical outcomes and therapeutic adherence, demonstrating the direct impact of applied HL in healthcare practice [29].

Among the articles included in this Special Issue, the study “Validation of the Adapted eHEALS Questionnaire for Assessing Digital Health Literacy in Social Media: A Pilot Study” [19] adapts the eHEALS scale to social media contexts, validating an instrument that assesses critical competencies for the purposes of interpreting online health information. The instrument demonstrated strong content validity and high internal consistency, supporting its suitability for population studies and the design of educational interventions to enhance DHL. This contribution is particularly relevant given the growing reliance on social media as a source of health information and the associated risks of misinformation.

However, HL also includes the ability to communicate clearly, empathetically, and educationally between professionals and patients. In this context, the article “Developing and Implementing a Narration of Care Framework to Teach Nurses When and How to Narrate Care” [21] addresses an identified gap in nursing education: the lack of structured guidance for explaining care to patients and families. Drawing on institutional data, patient satisfaction surveys (HCAHPS), and patient dialogue, the authors developed the RECAP framework (Removing uncertainty, Explaining the environment, Calm and sincere communication, Assume nothing, and Personal connection). Following its implementation across eight hospitals involving 7341 professionals, 99% of them reported improved ability to narrate care, demonstrating the framework’s value in systematising therapeutic communication and enhancing the patient experience.

Another relevant contribution addresses early academic training and professional commitment. The study “Effects of an Innovative Introductory Course on the Professional Commitment of First-Year Undergraduate Nursing Students: A Quasi-Experimental Study” [20] examines the impact of an introductory course on students’ professional commitment. Significant post-course improvements were observed, particularly in willingness to make an effort and adherence to professional values. Case-based learning and visits to healthcare centres were especially valued, indicating that well-designed early training can strengthen professional identity and support student retention.

The integration of environmental health into nursing education is addressed in the article “Spanish Nurses’ Knowledge and Perceptions of Climate Change: A Qualitative Study” [23]. While participants demonstrated awareness of climate-related health risks, the study identified barriers such as limited formal training, eco-anxiety, and insufficient educational resources. These findings underscore the urgency of incorporating planetary health as a core competence in nursing education.

Several studies in this Special Issue address mental HL. The article “Depression: [Mental] Health Literacy, Stigma, and Perceived Barriers to Help-Seeking During Transitions Among Undergraduate Nursing Students” [30] reports low recognition of depression and limited awareness of support resources among nursing students, underscoring the need to strengthen mental health content in curricula. Complementarily, “Understanding Family Experiences: A Study on Mental Health Literacy in Adolescent Eating Disorder Diagnoses” [24] examines families’ experiences during the diagnostic process and their levels of understanding, support, and capacity for action. Together, these findings highlight that health literacy must extend beyond the individual to include family contexts, support networks, and communities, thereby enhancing prevention, therapeutic adherence, and quality of life for patients and their families.

Emotional, ethical, and attitudinal literacy related to suicide is examined in the study “Evaluation of Attitudinal Beliefs Held by Medical and Nursing Students Towards Suicidal Behaviour” [31]. The findings reveal significant differences between groups: medical students reported more permissive attitudes toward suicide in terminally ill patients, whereas nursing students scored higher on beliefs related to suicide in general. These results underscore the urgent need to integrate targeted training on mental health, suicide prevention, ethical reasoning, and stigma reduction into health sciences education. Such training is essential not only for developing technical competence but also for fostering compassionate, reflective, and responsible professional practice, contributing to safer care environments and improved well-being for individuals at risk.

Conversely, the article “Moral Distress and Its Determinants among Nursing Students in an Italian University: A Cross-Sectional Study” [26] addresses moral distress among nursing students as both an educational concern and an ethical concern. The quantitative study found high levels of moral distress during clinical placements and academic activities, particularly when experiences conflicted with students’ ethical beliefs. Contributing factors included gaps between taught principles and clinical practice, limited resources, and insufficient institutional support for high-quality care. Higher distress levels were also reported among students who had not initially chosen nursing as their first career option, those combining work and study, and those who were separated or divorced, indicating that personal, academic, and contextual factors jointly increase vulnerability to ethical dilemmas. These findings underscore the need for nursing education programmes that systematically integrate content on professional ethics, resilience, and the management of moral distress. They also highlight the importance of providing structured opportunities for reflection, supervision, and emotional support to prepare future professionals not only in technical competencies but also in ethical sustainability, professional integrity, and psychosocial well-being.

Student satisfaction as an indicator of educational quality is examined in “Nursing Student Satisfaction Scale: Evaluation of Measurement Properties in Nursing Degree Programs” [24]. This study validated the Nursing Student Satisfaction Scale (NSSS) in the Italian context, supporting its usefulness for assessing key dimensions of the educational environment, including professional interaction, curriculum and teaching, and the learning context. The NSSS emerges as a reliable tool for evaluating and improving the educational experience, with potential implications for student retention and the quality of future nursing professionals.

Specific themes such as nursing knowledge and preparedness for rare diseases are also addressed. The study “Nurses’ Knowledge of Rare Diseases: A Systematic Review” [25] identifies substantial training gaps that affect care quality, highlighting the need for continuing education and technological resources to support care for patients with complex needs. In the area of maternal and perinatal HL, the study “Health Literacy in Pregnant Women and Its Associations with Personal, Socioeconomic, and Health-Related Factors in Primary Care” [26] examines HL levels among pregnant women and their associations with personal, socioeconomic, and health-related factors in primary care. The findings reveal marked variability across social determinants, underscoring the need for prenatal care to account for these differences to ensure effective communication, promote adherence to preventive care, and support informed decision-making. Overall, this evidence underscores the importance of assessing HL in vulnerable populations and adapting prenatal health education to reduce inequalities and improve maternal and child outcomes.

Ageism and its determinants in healthcare professionals are analysed in “Ageism and Associated Factors in Healthcare Workers: A Systematic Review” [27]. The review shows that healthcare workers may hold ageist attitudes and prejudices, which are influenced by individual and work-related factors, including limited gerontological training, limited experience in geriatric settings, and demanding working conditions. Although overall levels of ageism were generally low, substantial variability across contexts was observed, along with a consistent association between greater geriatric training or experience and more positive attitudes towards older adults. These findings highlight the importance of incorporating targeted gerontological education, promoting supervised intergenerational contact, and addressing organisational factors to reduce prejudice and improve care quality for ageing populations. Integrating these measures into nursing education also supports HL for older adults by adapting communication, educational materials, and interventions to their needs, ultimately fostering more equitable and person-centred care.

Finally, the practical application of HL is illustrated in the “Propensity Score Analysis of the Utility of Supervised Perioperative Abdominal Wall Exercises for the Prevention of Parastomal Hernia” [28], which evaluates the effectiveness of a supervised abdominal wall exercise programme implemented before and after stoma creation. The results showed a significant reduction in the risk of parastomal hernia among participants performing supervised exercises. These findings highlight the value of incorporating structured, nurse- and physiotherapist-coordinated physiotherapy routines as an integral component of perioperative care, thereby improving surgical outcomes and enhancing long-term quality of life.

Together, the studies in this Special Issue show that HL and DHL are cross-cutting constructs spanning multiple dimensions of nursing education and practice. Although they address diverse topics—including digital literacy assessment using validated instruments [19], care narration and therapeutic communication [21], early training and professional commitment [20], mental health and stigma reduction [30,31], ethics and moral distress [26], climate change and environmental health [23], ageing and ageism [27], and the application of educational programmes to prevent clinical complications [29] —they all share a common focus: developing the capacity to access, understand, critically evaluate, and apply health information in complex, digital, and socially diverse contexts, as described in core HL frameworks [3,4]. From this perspective, HL and DHL extend beyond information transmission and are conceptualised as professional competencies integrating communicative, digital, ethical, and relational skills essential for shared decision-making, person-centred care, and the reduction in health inequalities [17].

This monograph aims to contribute to reflection and the development of strategies that promote HL as a driver of transformation in nursing education and practice, fostering professionals better prepared to address current and future health system challenges and to respond effectively to the needs of individuals and communities.

In conclusion, this Special Issue reinforces the need to conceptualise HL and DHL as core competencies of contemporary nursing, with direct implications for education, clinical practice, and research. Future training initiatives and studies should be grounded in consolidated theoretical frameworks, including integrated HL models [3]; Nutbeam’s distinction between functional, interactive, and critical literacy [32,33]; person-centred care approaches; and DHL models applied to digital environments and social media [4,19]. Available evidence supports the implementation of explicit, longitudinal, and assessable programmes that combine active methodologies, innovative learning environments, and training in clear communication, critical thinking, and responsible use of digital resources [14]. Integrating these strategies can enhance nurses’ professional competencies, strengthen patient safety, and contribute to more equitable, resilient, and humanised health systems [17].
